# Evaluation of prophylactic efficacy of cinnamaldehyde in murine model against *Paradendryphiella arenariae* mycotoxin tenuazonic acid-induced oxidative stress and organ toxicity

**DOI:** 10.1038/s41598-021-98319-8

**Published:** 2021-09-30

**Authors:** Ankita Kumari, Karuna Singh

**Affiliations:** Animal Mycology Laboratory, Department of Zoology, Mahila Mahavidyalaya, Banaras Hindu University, Varanasi, 221005 India

**Keywords:** Biochemistry, Microbiology, Zoology

## Abstract

Cinnamaldehyde (Cin) is a natural product obtained from cinnamon and is reported to have a potential anti-fungal, anti-oxidant, anti-inflammatory and anticancer effect. The present study investigated the possible protective role of Cin against tenuazonic acid-induced mycotoxicity in the murine model. Tenuazonic acid (TeA), a toxin produced by *Alternaria* is a common contaminant in tomato and tomato-based products. Here, Swiss male mice were administered with TeA isolated from *Paradendryphiella arenariae* (MW504999) (source-tomato) through injection (238 µg/kg BW) and ingestion (475 µg/kg BW) routes for 2 weeks. Thereafter, the prophylaxis groups were treated with Cin (210 mg/kg BW). The experiment was carried out for 8 weeks. The treated groups were compared to the oral and intra-peritoneal experimental groups that received the toxin solely for 8 weeks. Haematological, histopathological and biochemical aspects of the experimental and the control mice were analysed. Sub-chronic intoxication of mice with TeA showed elevated malondialdehyde (MDA), reduced catalase (CAT) and superoxide dismutase (SOD) production; abnormal levels of aspartate transaminase (AST) and alanine transaminase (ALT). Treatment with Cin reversed TeA-induced alterations of antioxidant defense enzyme activities and significantly prevented TeA-induced organ damage. Thus, cinnamaldehyde showed therapeutic effects and toxicity reduction in TeA induced mycotoxicosis.

## Introduction

Tenuazonic acid (TeA) is the most important *Alternaria* mycotoxin and is regarded as extremely toxic^1,2^. It is predominantly present in tomatoes, apples and the commodities like beer and cereal foods^3^. Chemically, TeA is a tetrameric derivative and an amide metabolite. The toxic effects of TeA are attributed to its ability to inhibit newly formed proteins from the ribosome^4^. It has been tested in chick embryos, mice, guinea pigs, rabbits, dogs and rhesus monkeys and its effects involve cardiovascular collapse and gastrointestinal haemorrhage^2,5^. Historically, TeA has been associated with a haematological disorder named Onyalai that occurred in central and southern Africa^6^. Tenuazonic acid has been reported to cause emesis, salivation, tachycardia, hemorrhages and hemorrhagic gastro-enteropathy in rats, mice, dogs and monkeys^7^. Sub-lethal to lethal doses of TeA in the feed caused haemorrhages in several organs and thigh muscles of the egg-laying hens and broiler chickens^8^.

Oxidative stress (OS) has been stated as the major reason behind the mycotoxicity leading to numerous other effects. The generation of reactive oxygen species (ROS) leads to peroxidative damage to the vital organs^9^. Mycotoxins provoke toxicity by producing cytotoxic effects and ROS. Under normal conditions, ROS has a vital function in cell signalling and homeostasis. It also plays an important role in apoptosis^10^. Elevated mycotoxin concentration for a long duration leads to an increase in the ROS and MDA; hence decreasing cell viability. Mechanism of illness caused by mycotoxins includes inflammation, oxidative stress, toxicity infection, allergy and exposure-induced irritant effects^11^.

There is no possible treatment for mycotoxin exposure except for supportive therapy (e.g., diet, hydration)^12^. However, a combination of additives like ammonia, propionic acid, and microbial enzymatic silage additives has been shown to inhibit the growth of mould^13^.

Natural products have been used to treat health ailments for decades. Leaves and barks of *Cinnamamum zeylanicum* (Cz) were demonstrated to exhibit potential antimould activity against *Alternaria solani* and *Curvularia lunata*^14^. Cinnamaldehyde is an active ingredient of cinnamon which is obtained from the trees of *Cinnamamum* sps. The antifungal activity of Cin has already been reported against *Cryptococcus neoformans* and filamentous fungi^14,15^. Cin also exhibits anti-fungal activity against *Geotrichum citri-aurantii*^16^.

The present study is, therefore, an endeavor to explore the potential usefulness of Cin in reducing TeA induced sub-chronic mycotoxicity. The oxidative stress resistance by cinnamaldehyde in mice model induced with TeA toxicity has been investigated. Usually, toxicological studies are focused on the liver and kidneys where the metabolism and excretion of the toxin takes place. But in the current study, all the vital organs have been taken into account to get a complete set of information on the effect and the target organ of the toxin.

## Results

### Route of exposure of TeA and Cin treatment efficacy

TeA was found to be more toxic when administered through intra-peritoneal (IP) route than the oral (PO) route. Oral treatment of Cin at a concentration of 210 mg/kg/day BW was found to be most effective in both the treatment groups (POT-oral administration of TeA and oral administration Cin treatment and IPT-Intra-peritoneal administration of TeA and oral administration Cin treatment). At high concentration (420 mg/kg/day BW), mice died at the 3rd and 4th day of treatment while at a low concentration (105 mg/kg/day BW) prophylaxis was not effective.

### Change in weight and feed consumption

**Figure 1:**
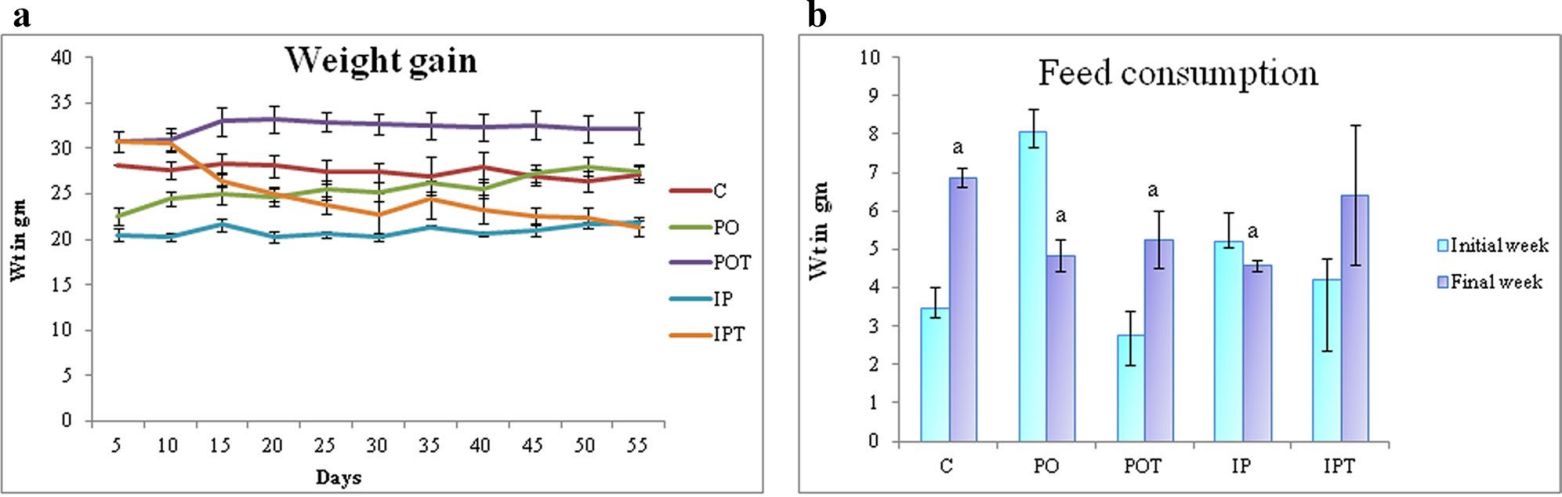
(**a**) Comparison of effect of the TeA and Cin treatment on weight gain of mice over 8 weeks. (**b**) Comparison of the effect of TeA and Cin treatment on feed consumption in the initial and final week of experiment. The results are shown in mean ± SE (‘a’ represents p < 0.005 initial vs final week). Wt in gm is weight in grams.

Changes were observed in the performance and health of mice during the experimental period. The growth performance of mice was affected, since the mice of the mycotoxicosis induced (MI) group showed higher weight gain and lower feed consumption while that of the prophylaxis group showed lower weight gain and higher feed consumption like the control group (Fig. 1a,b).

Considering the weight gain of the mice during the entire period of experimentation, it was observed that a slight increase in weight of the mycotoxicosis induced (MI) group i.e., [PO (green), IP (blue)] took place. Both the groups showed a similar trend of weight gain. On the contrary, the prophylactic groups [POT (purple), IPT (orange)] showed a different trend. The POT group showed no change in weight gain like the control group (red) (Fig. 1a). While the IPT group, showed a significant decrease in the weight gain over the period. On observing the weights of the mice in the initial and final weeks of the experiment, a significant increase in both PO and IP groups was noted. The prophylactic group POT showed no change in weight while the IPT group showed a significant decrease in weight of mice (Fig. 1a).

### Relative organ weight

**Figure 2:**
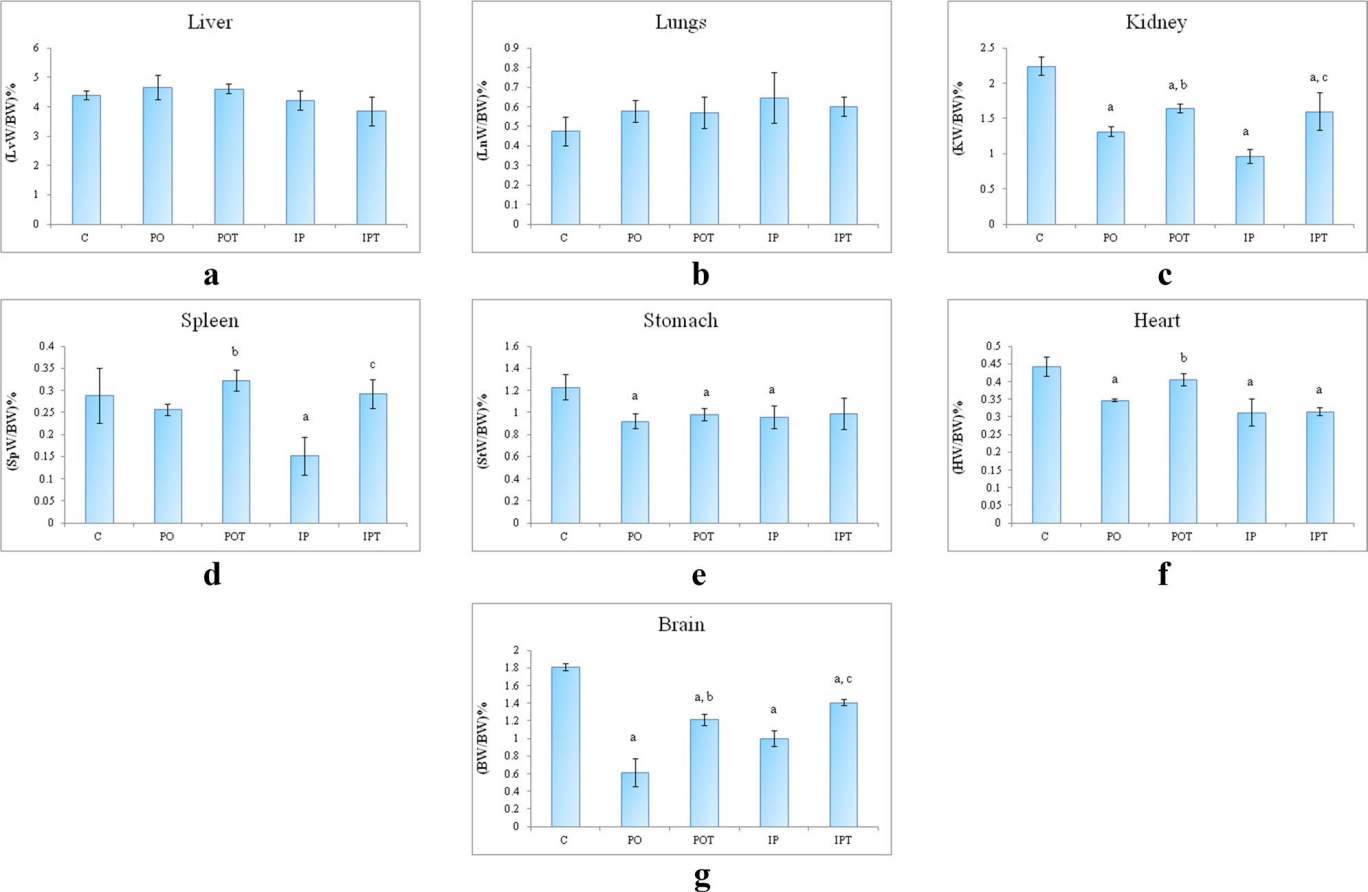
Relative organ: body weight ratio of (**a**) liver, (LvW/BW is weight of liver/body weight)%, (**b**) lungs, (LnW/BW is weight of lungs/body weight)%, (**c**) kidney, (KW/BW is weight of kidneys/body weight)%, (**d**) spleen, (SpW/BW is weight of spleen/body weight)%, (**e**) stomach, (StW/BW is weight of stomach/body weight)%, (**f**) heart, (HW/BW is weight of heart/body weight)%, (**g**) brain, (BW/BW is weight of brain/body weight)%. The results are shown in mean ± SE (‘a’ represents p < 0.005 control vs experimental groups, ‘b’ represents p < 0.005 experimental vs treatment groups (PO vs POT) and ‘c’ symbolizes p < 0.005 IP vs IPT). The x-axis comprises of the different groups—control (C), oral toxin (PO), oral toxin and oral Cin treatment (POT), intra-peritoneal toxin (IP), intra-peritoneal toxin and oral Cin treatment (IPT).

No significant changes were observed in the relative weight of the liver of PO and IP (mycotoxicosis induced) as well as the POT and IPT (treatment) groups (Fig. 2a). The lungs of the mycotoxicosis induced groups showed a slight increase in weight but the change was not significant in comparison to the control (C) group. The prophylactic group again showed no change in the weight of lungs: body weight indices (Fig. 2b). In comparison to the control group (Fig. 2c); the weight of kidneys of both the mycotoxicosis induced groups dropped significantly. In the prophylactic groups, the weight of kidneys was close to normal but still lower than the control group. A significant reduction in the weights of the spleen of the mycotoxicosis induced groups was observed (Fig. 2d). The higher spleen: body weight indices of prophylactic groups indicated immunomodulation. The stomach: body weight indices declined in the PO, IP and POT groups (Fig. 2e). But the weight of the IPT group remained at par with the control group. The weight of heart of both the MI group and IPT treatment groups showed significantly low values. The weight of heart was similar to control in the POT group (Fig. 2f). The brain of the experimental (PO, IP) groups showed a significant lowering in weight. The brain: body weight ratio increased in the prophylactic groups (POT, IPT) when compared to their respective mycotoxicosis groups (Fig. 2g) but in comparison to the control group, the weight remained low.

### Circulating WBCs

**Figure 3:**
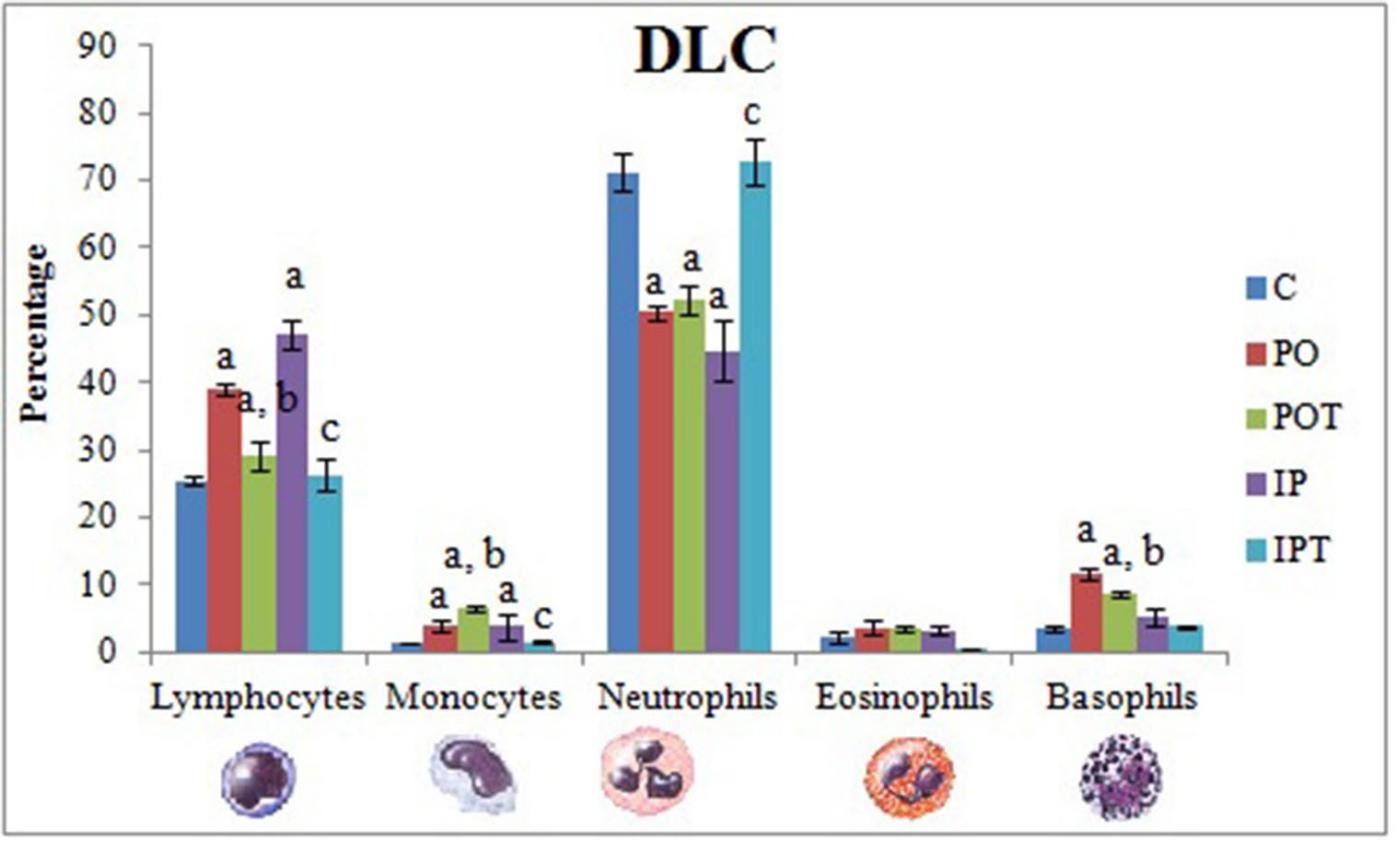
Differential leukocyte count comparing the percentage of WBCs in different groups. The results are shown in mean ± SE (‘a’ symbolizes p < 0.005 control vs experimental groups, ‘b’ symbolizes p < 0.005 experimental vs treatment groups (PO vs POT) and ‘c’ symbolizes p < 0.005 IP vs IPT).

The differential leukocyte count results showed signs of allergic reaction in the case of MI group. Both PO and IP groups revealed elevated lymphocytes, monocytes and basophils percentage which could not revert to normal in the Cin treated group-POT (Fig. 3). The IP and PO groups also showed neutropenia indicating signs of infection which became normal in the Cin treated group (IPT).

### Biochemical analyses and histopathology (Organ wise)

#### Liver

**Figure 4:**
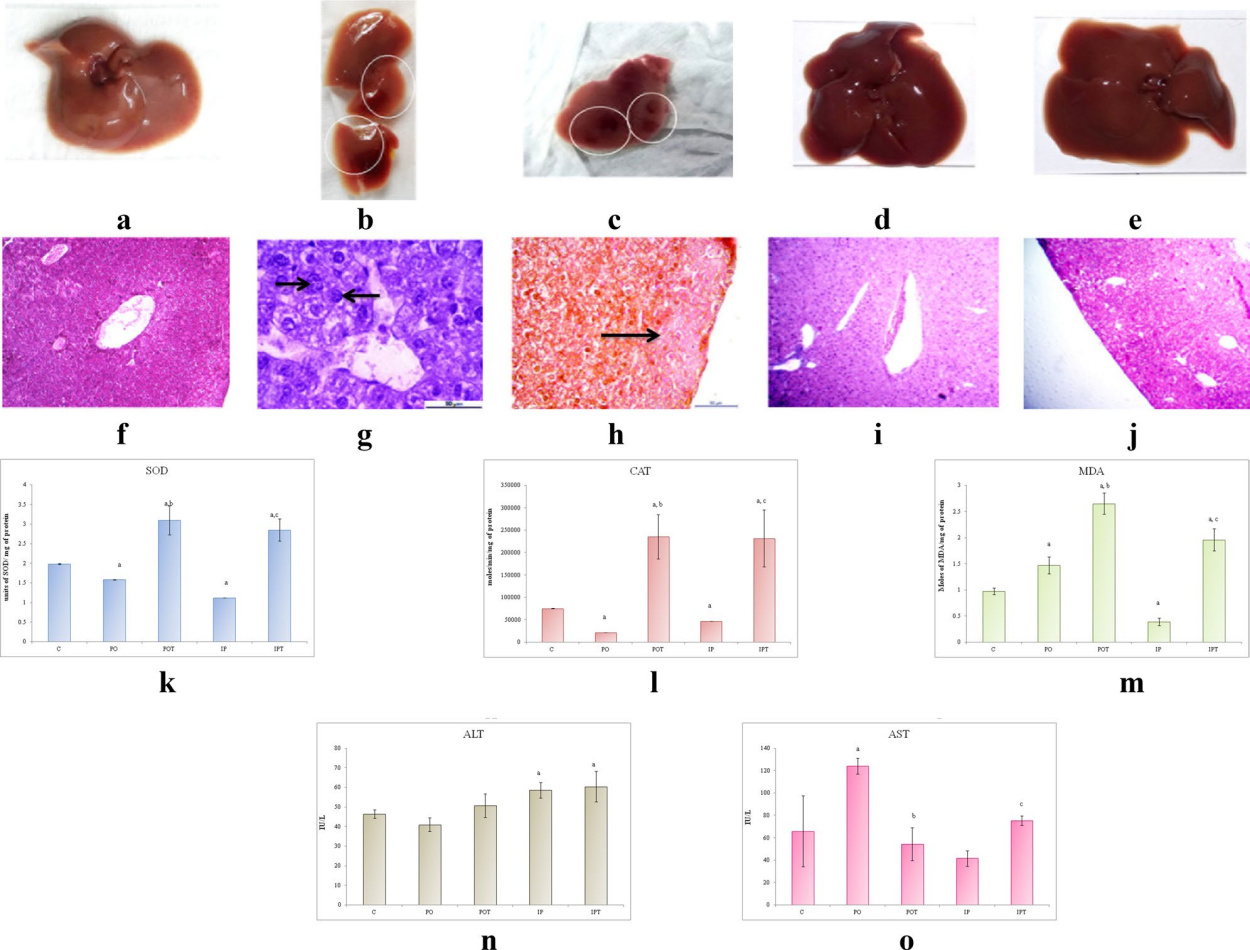
Photographs of liver of (**a**) control, (**b**) PO group showing discolouration as indicated by circles, (**c**) IP group showing gross lesions as indicated by circles, (**d**) POT treatment group with no pathological changes, (**e**) IPT group with no pathological changes. Photomicrographs of TS of liver of (**f**) control (HE, ×100), (**g**) PO group (HE, ×400) showing karyomegaly. Arrow indicates enlargement of hepatocytes and nucleus (HE, ×400) (**h**) IP group (HE, ×100) showing hemorrhagic necrosis (arrow) (**i**) POT-treated group, showing normal histology (H/E, ×100), (**j**) IPT-treated group (HE, ×100). (**k**) Comparison of SOD activity in liver of the control, experimental and treated animals. (**l**) Comparison of CAT activity in liver of control, experimental and treated animals. (**m**) Comparison of MDA activity in liver of control, experimental and treated animals. (**n**) Comparison of ALT activity in liver of control, experimental and treated animals. (**o**) Comparison of AST activity in liver of control, experimental and treated animals. The results of biochemical assays are shown in mean ± SE (‘a’ symbolizes p < 0.005 control vs experimental groups, ‘b’ symbolizes p < 0.005 experimental vs treatment groups (PO vs POT) and ‘c’ symbolizes p < 0.005 IP vs IPT).

The size and weight of the liver of both the MI group was found to be equal to that of the control group (Fig. 4a–c). The liver also showed discoloration (PO) (Fig. 4b) and gross lesions (IP) (Fig. 4c) on autopsy. No signs of pathology were seen in the treatment groups (POT, IPT, Fig. 4d,e). Unlike control (Fig. 4f), the histological sections of the PO group revealed karyomegaly in the portal triad region (Fig. 4g) whereas haemorrhagic necrosis was observed in the liver parenchyma of IP group (Fig. 4h). The histology of the treatment groups exhibited normal architecture of cells (POT, IPT; Fig. 4i,j).

The antioxidant enzymes, SOD and CAT showed a decline in the mycotoxicosis induced groups (Fig. 4k,l). An elevation in MDA level was noted in the PO group but in case of IP group, it was found to decline (Fig. 4m). The increased levels of SOD, CAT and MDA enzymes were observed in the prophylaxis group (Fig. 4k–m).

The liver function test presented higher levels of ALT (in IP group) and AST (in PO group) and low levels of ALT (in PO group) and AST (in IP group). The treatment group normalised both the ALT and AST except for the level of ALT in the IPT group which augmented even after treatment (Fig. 4n,o).

#### Kidney

**Figure 5:**
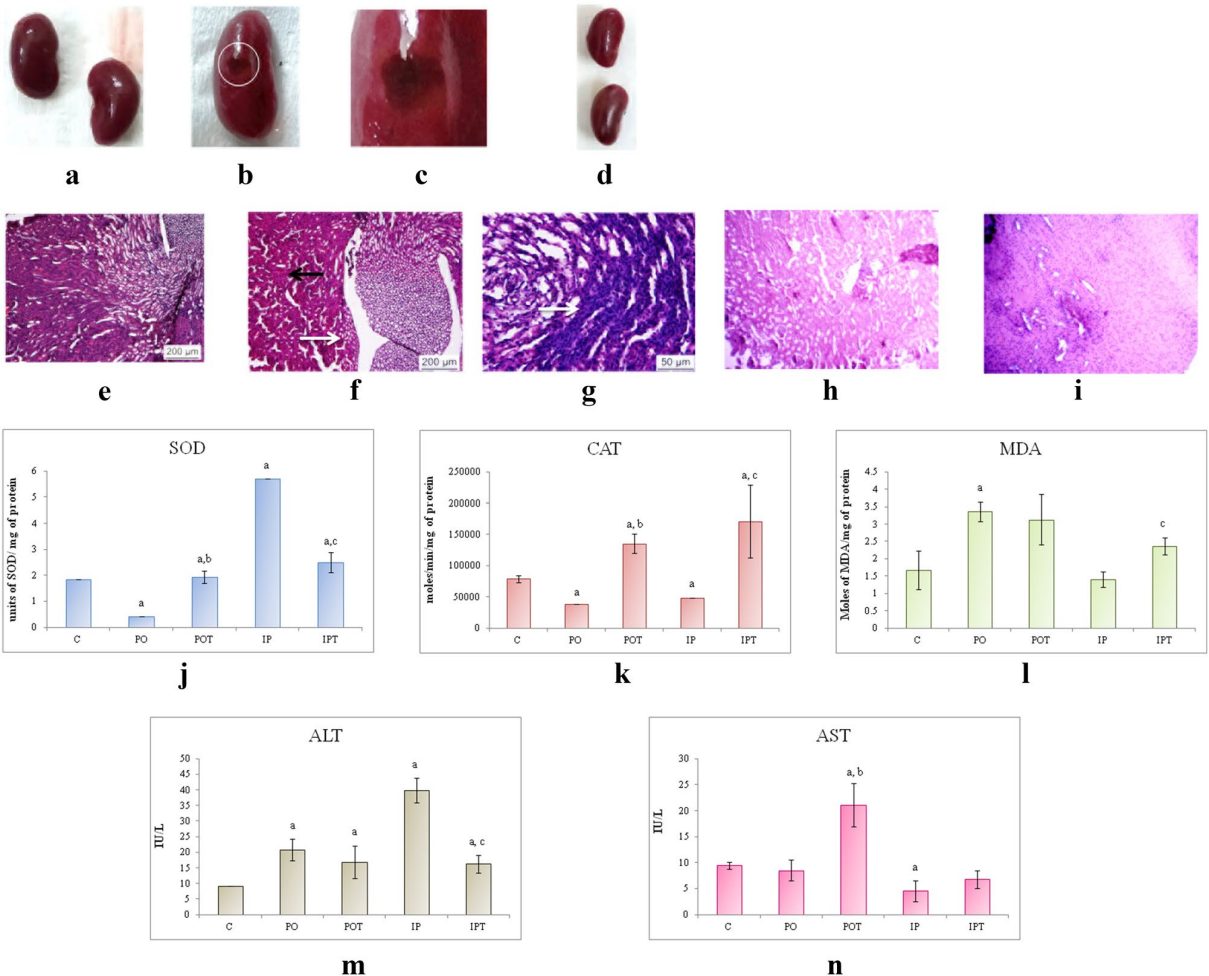
Photographs of kidney of (**a**) control, (**b**) IP group showing gross lesions as indicated by a circle, (**c**) enlarged view of the lesion, (**d**) treatment group with no pathological changes. Photomicrographs of TS of kidney, (**e**) control (HE, ×100), (**f**) PO-showing vacuolization (white arrow) and haemorrhage (black arrow) in the medulla region (HE, ×100), (**g**) IP-showing infiltration in cortical region (HE, ×400), (**h**) POT-treated mouse showing no pathology (HE, ×100), (**i**) IPT-treated mouse showing no pathology (HE, ×100). (**j**) Comparison of SOD activity in the kidney of control, experimental and treated animals. (**k**) Comparison of CAT activity in the kidney of control, experimental and treated animals. (**l**) Comparison of MDA activity in the kidney of control, experimental and treated animals. (**m**) Comparison of ALT activity in the kidney of control, experimental and treated animals. (**n**) Comparison of AST activity in the kidney of control, experimental and treated animals. The results of biochemical assays are shown in mean ± SE (‘a’ symbolizes p < 0.005 control vs experimental groups, ‘b’ symbolizes p < 0.005 experimental vs treatment groups (PO vs POT) and ‘c’ symbolizes p < 0.005 IP vs IPT).

On autopsy, kidney of the control mice (Fig. 5a) showed normal morphology while IP group exhibited a gross lesion on the cortical region (Fig. 5b,c). The kidneys of the treated mice (Fig. 5d) showed no such changes. Unlike control (Fig. 5e), the signs of vacuolization in the cortical region and haemorrhage in the interstitium were observed in the renal histology of PO group (Fig. 5f). However, histology of IP group showed leukocyte infiltration in the medullary region (Fig. 5g). No pathological changes were noted in the prophylactic groups (Fig. 5h,i).

The levels of SOD and CAT were lowered in MI group except for SOD in the IP group which was very high (Fig. 5j,k). MDA assay showed elevated levels in PO while low levels in the IP group (Fig. 5l). The treatment groups restored the level of enzymes near to the control group. CAT in the treatment groups was higher as compared to the control. An increase in ALT was noted in both the MI groups while its normal level was restored in the treatment group (Fig. 5m). No change was observed in the AST enzyme in the PO group but a significant decline in its level was recorded in the IP group. The treatment group (POT) showed high levels of the AST (Fig. 5n). The levels of antioxidant enzymes suggested oxidative stress.

#### Stomach

**Figure 6:**
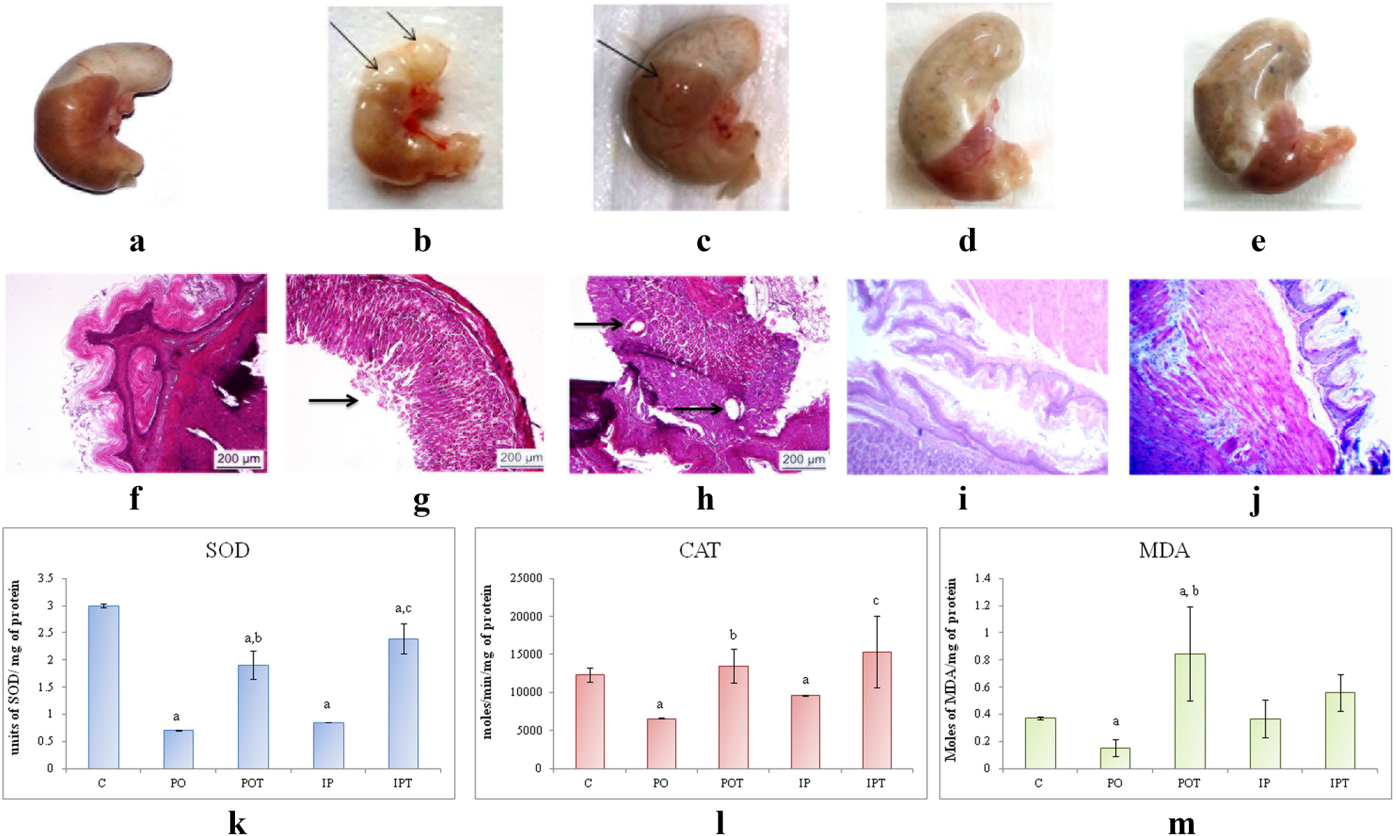
Stomach of (**a**) control, (**b**) PO group showing presence of multiple tumours as indicated by arrows, (**c**) IP group showing presence of tumour indicated by an arrow, (**d**) POT treatment showing normal stomach, (**e**) IPT group with no pathological changes. Photomicrographs of TS of stomach (**f**) control (HE, ×100) (**g**) PO-showing vacuolization (HE, ×100) (**h**) IP-showing ulcer (HE, ×100) (**i**) POT-showing normal architecture (HE, ×100) (**j**) IPT-showing normal architecture (HE, ×100). (**k**) Comparison of SOD activity in stomach of the control, experimental and treated animals. (**l**) Comparison of CAT activity in stomach of the control, experimental and treated animals. (**m**) Comparison of MDA activity in stomach of the control, experimental and treated animals. The results of biochemical assay are shown in mean ± SE (‘a’ symbolizes p < 0.005 control vs experimental groups, ‘b’ symbolizes p < 0.005 experimental vs treatment groups (PO vs POT) and ‘c’ symbolizes p < 0.005 IP vs IPT).

Macromorphological observation of the stomach showed presence of multiple tumours when compared to the stomach of control animal (Fig. 6a–c) which was further confirmed by the presence of metastatic cells/tissues in histological sections (HE staining). Tumours were observed in the forestomach of the PO group (Fig. 6b) and in between the forestomach and the corpus in the IP group (Fig. 6c). Cin treatment or prophylaxis groups showed no such pathology (Fig. 6d,e). As compared to the control (Fig. 6f), the histology of PO stomach showed ulcers in the lamina propria (Fig. 6g) whereas the IP group showed vacuolization in the simple columnar epithelium glandular cell lining of mucosa (Fig. 6h). Sections of both the MI groups show atypical hyperplasia (Fig. 6g,h). Histology of the stomach of prophylaxis group appeared normal which was confirmed by the histology (Fig. 6i,j) and antioxidant assays (Fig. 6k–m).

A detailed analysis of the antioxidant enzymes showed decreased SOD and CAT activities in both the MI groups (Fig. 6k,l). MDA in the IP group remained almost equal to that of the control group (Fig. 6m). Cin showed a protective role against TeA induced tumours in the stomach, and thus elevated the activity of SOD, CAT and LPO in prophylaxis groups.

#### Spleen

**Figure 7:**
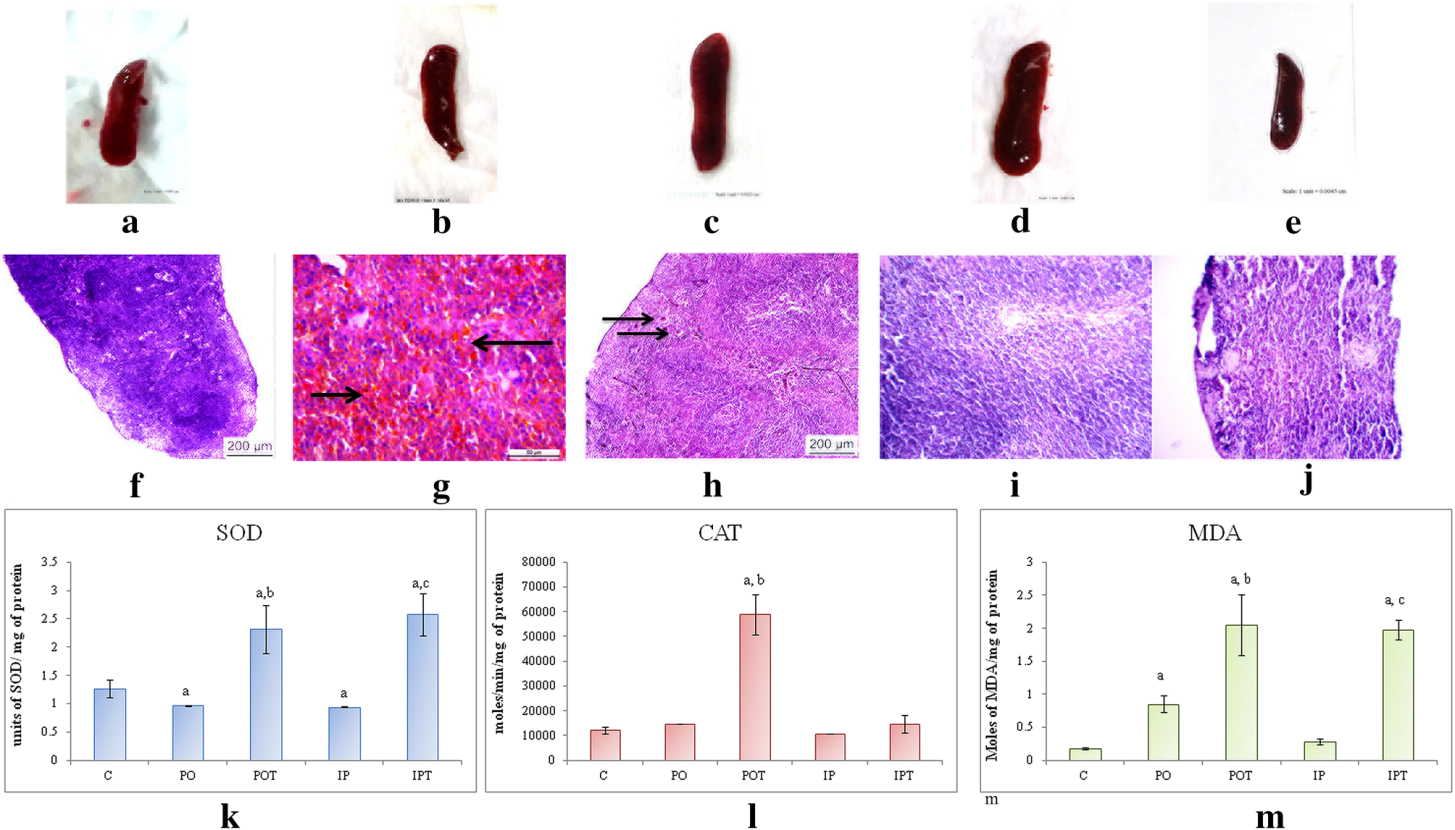
Spleen of (**a**) control, (**b**) PO group, (**c**) IP group, (**d**) POT treatment, (**e**) IPT group. Photomicrographs of TS of spleen (**f**) control (HE, ×100) (**g**) PO-showing apoptotic cells (arrows) (HE, ×100) (**h**) IP-showing haemorrhage (HE, ×400) (**i**) POT-showing no pathological changes (HE, ×100) (**j**) IPT-showing normal architecture of the cells (HE, ×100). (**k**) Comparison of SOD activity in spleen of the control, experimental and treated animals. (**l**) Comparison of CAT activity in spleen of the control, experimental and treated animals. (**m**) Comparison of MDA activity in spleen of the control, experimental and treated animals. The results of biochemical assays and spleen size are shown in mean ± SE (‘a’ symbolizes p < 0.005 control vs experimental groups, ‘b’ symbolizes p < 0.005 experimental vs treatment groups (PO vs POT) and ‘c’ symbolizes p < 0.005 IP vs IPT).

On morphological observation, the weight of spleen of the MI group were notably lower than that of the control group (Fig. 7a–c) while these changes were not seen in the prophylaxis group (Fig. 7d,e). Unlike control group (Fig. 7f), the signs of hyperplasia (Fig. 7g) and haemorrhage (Fig. 7h) were noticed in the PO and IP groups respectively. The histology of the prophylaxis groups (Fig. 7i,j) appeared as normal as control.

Low values of SOD and CAT (not significant, Fig. 7k,l) and higher activity of MDA (Fig. 7m) indicated oxidative stress. The condition was restored to normal under the action of Cin. SOD in both IPT and POT increased from 0.97 (PO) to 2.32 (POT) and 0.94 (IP) to 2.58 (IPT) units of SOD/mg of protein. CAT level in POT was very high as compared to the control. Significantly high activity of MDA was also recorded in the IPT and POT groups, again much higher than the control.

#### Lungs

**Figure 8:**
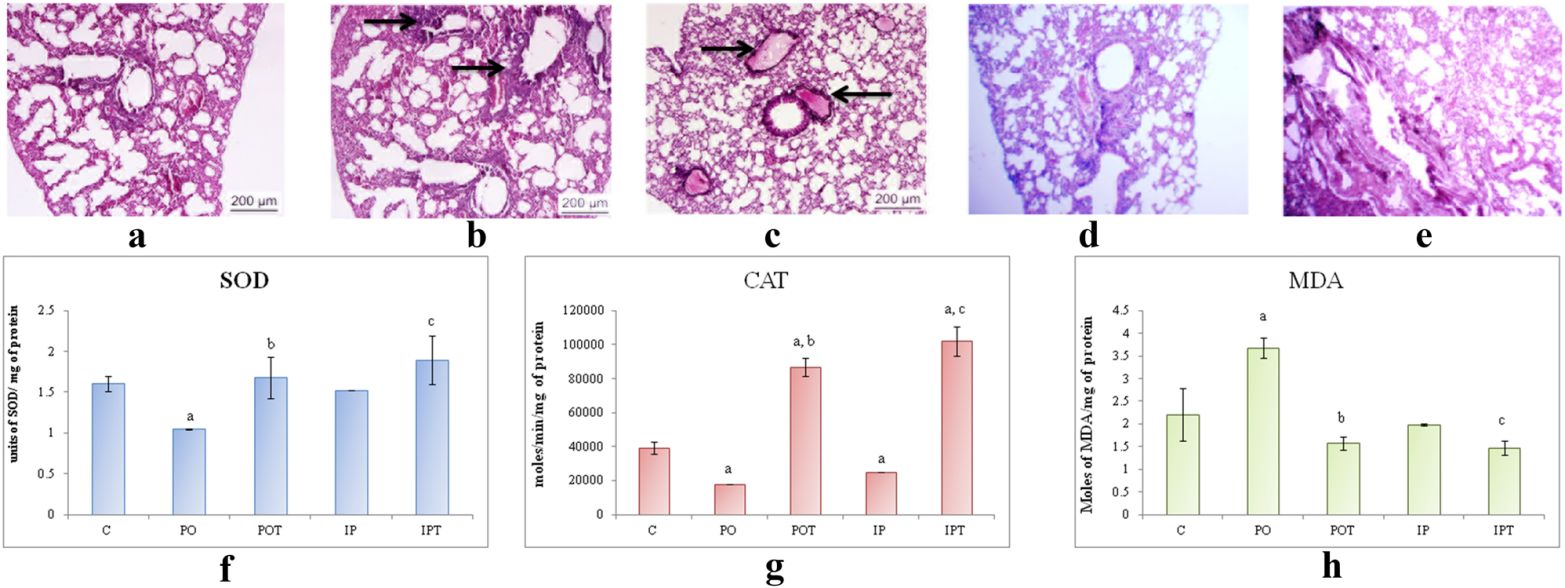
Photomicrographs of TS of lung of (**a**) control (HE, ×100) (**b**) PO-experimental mouse showing inflammation around the bronchiole (HE ×100) (**c**) IP-experimental mouse showing pulmonary oedema in the alveoli (HE, ×100) (**d**) POT-treated mouse (HE, ×100) showing no pathological condition (HE, ×100) (**e**) IPT-treated mouse showing no pathological condition (HE, ×100). (**f**) Comparison of SOD activity in lungs of the control, experimental and treated animals. (**g**) Comparison of CAT activity in lungs of the control, experimental and treated animals. (**h**) Comparison of MDA activity in lungs of the control, experimental and treated animals. The results of biochemical assays are shown in mean ± SE (‘a’ symbolizes p < 0.005 control vs experimental groups, ‘b’ symbolizes p < 0.005 experimental vs treatment groups (PO vs POT) and ‘c’ symbolizes p < 0.005 IP vs IPT).

On autopsy, the lungs appeared normal. The histology of the lungs, however, showed oedema and inflammation in the IP and PO groups respectively (Fig. 8a–c). The lungs of treated (Fig. 8d,e) groups appeared normal. The SOD and CAT activities in the MI groups were less than that of the control group but were very high in the IPT and POT groups (Fig. 8f,g).

The results of the MDA showed different trends in both the routes of administration (Fig. 8h). The intraperitoneal route showed a slight decrease in MDA activity which further decreased on treatment. On the contrary, MDA activity was very high in the oral route which decreased after Cin treatment.

#### Heart

**Figure 9:**
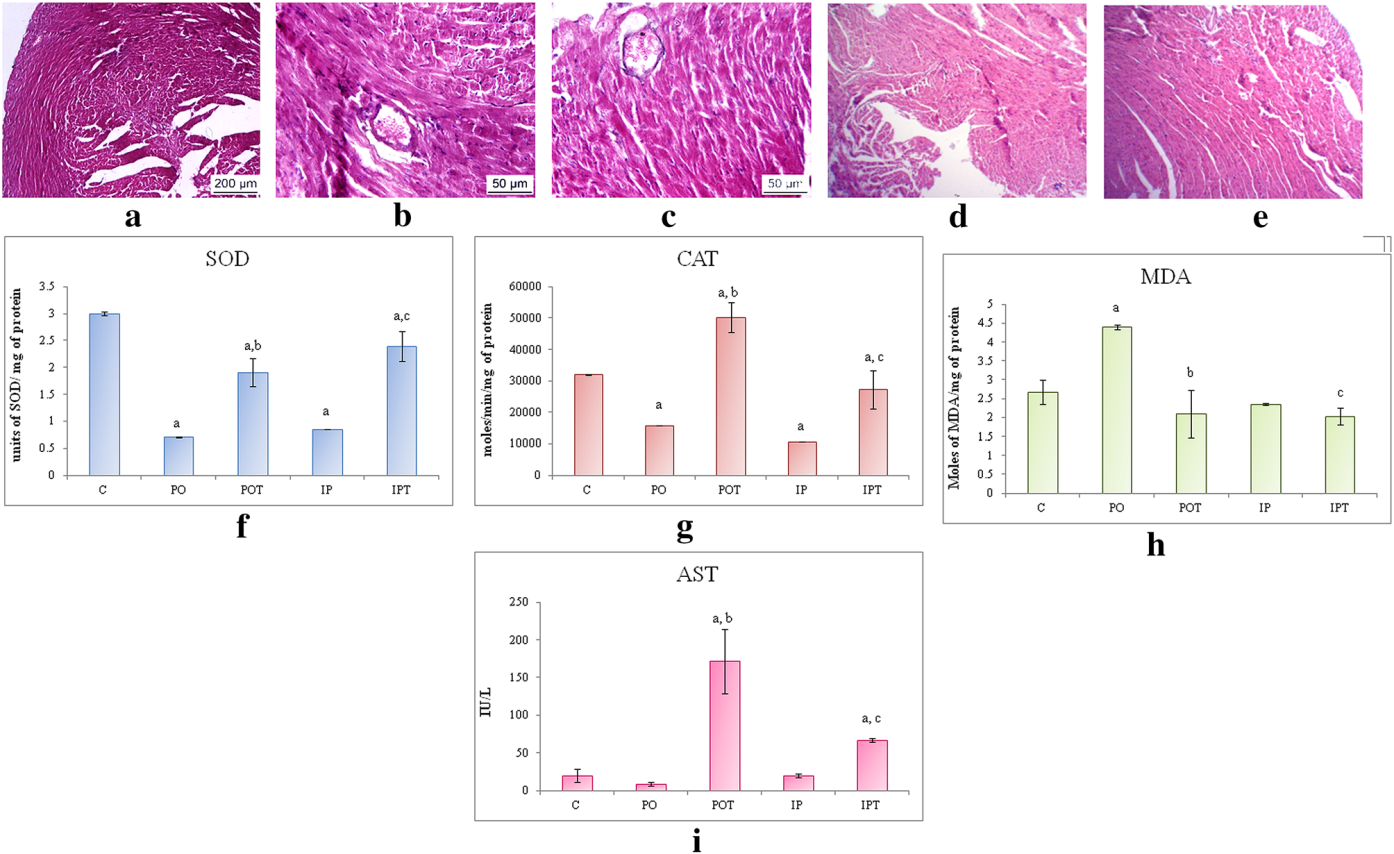
Photomicrographs of TS of heart of (**a**) control (HE, ×100) (**b**) PO-showing shortening and thickening of the tendinous cords (HE, ×400) (**d**) IP-showing cyst and haemorrhage (HE, ×400) (**c**) POT-showing normal cardiac tissues (HE, ×100) (**e**) IPT-showing normal cardiac tissues (HE, ×100). (**f**) Comparison of SOD activity in heart of the control, experimental and treated animals. (**g**) Comparison of CAT activity in heart of the control, experimental and treated animals. (**h**) Comparison of MDA activity in heart of the control, experimental and treated animals. (**i**) Comparison of AST activity in heart of the control, experimental and treated animals. The results of biochemical assays are shown in mean ± SE (‘a’ symbolizes p < 0.005 control vs experimental groups, ‘b’ symbolizes p < 0.005 experimental vs treatment groups (PO vs POT) and ‘c’ symbolizes p < 0.005 IP vs IPT).

The histological section showed cyst and haemorrhage in the ventricular region of heart of MI group when compared to the control group (Fig. 9a–c). The inflammatory cells were also observed in the tissue around the cyst. The Cin treatment maintained the normal architecture of cardiac tissues and the levels of all the three enzymes that were examined (Fig. 9d,e).

ROS studies were in agreement with the above results. The histology showed that TeA affected the heart as well. Low SOD, CAT and high MDA showed that TeA stimulated OS (Fig. 9f–h). Notably high values of AST were recorded in the prophylaxis group while changes in the MI groups were not significant (Fig. 9i).

#### Brain

**Figure 10:**
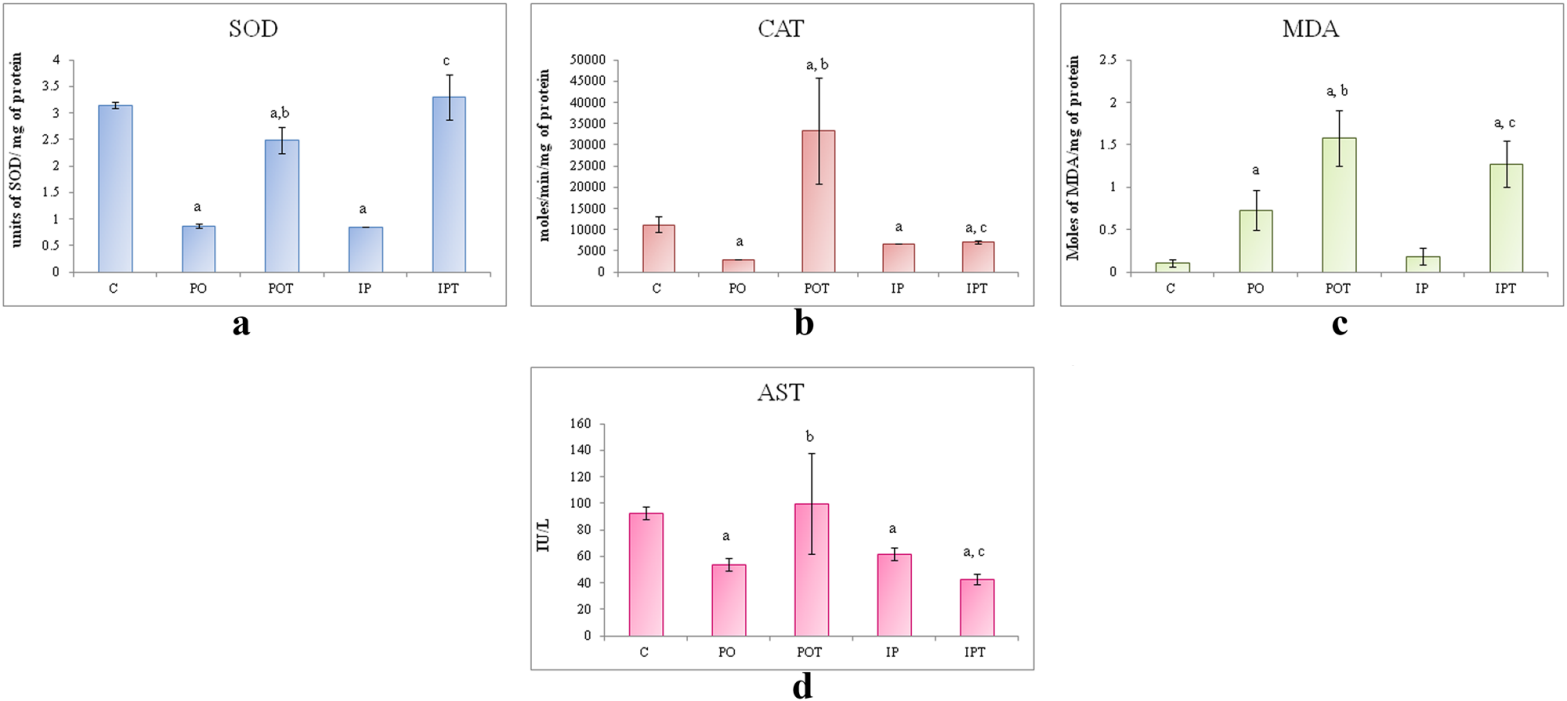
(**a**) Comparison of SOD activity in brain of the control, experimental and treated animals. (**b**) Comparison of CAT activity in brain of the control, experimental and treated animals. (**c**) Comparison of MDA activity in brain of the control, experimental and treated animals. (**d**) Comparison of AST activity in brain of the control, experimental and treated animals. The results of biochemical assays are shown in mean ± SE (‘a’ symbolizes p < 0.005 control vs experimental groups, ‘b’ symbolizes p < 0.005 experimental vs treatment groups (PO vs POT) and ‘c’ symbolizes p < 0.005 IP vs IPT).

TeA lowered the activity of both SOD (Fig. 10a) and CAT (Fig. 10b) whereas LPO (Fig. 10c) was profoundly increased in the brain. The prophylactic groups showed an increase in the activity of all the three enzymes. Rendering to the anti-oxidant effect of cinnamaldehyde, the oxidative stress in the MI groups was found to be notably reduced. The AST enzyme decreased in both the MI groups. Its concentration was high in the POT but was significantly low in the IPT group (Fig. 10d).

Histology of brain did not show any pathological changes and therefore, has not been presented here.

### Cell apoptosis factor

**Figure 11:**
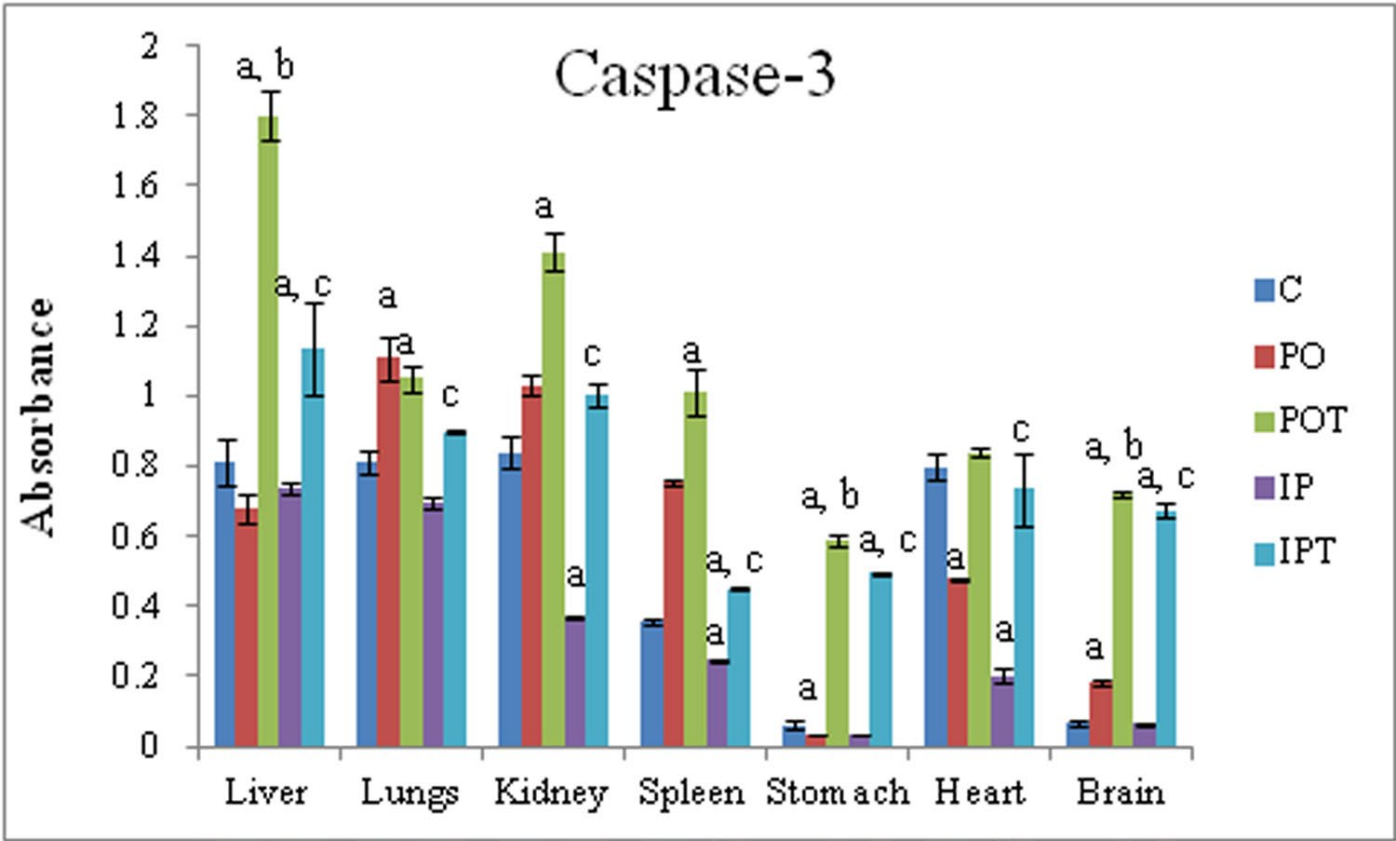
Comparison of effect of the TeA (experimental groups) and Cin (treatment groups) on the Caspase-3 enzyme in different organs. The results are shown in mean ± SE (‘a’ symbolizes p < 0.005 control vs experimental groups, ‘b’ symbolizes p < 0.005 experimental vs treatment groups (PO vs POT) and ‘c’ symbolizes p < 0.005 IP vs IPT).

The mycotoxicosis induced groups showed noticeably low levels of caspase-3 in the liver, lungs, kidney, spleen, stomach, heart and brain (Fig. 11) which increased significantly in the prophylaxis groups.

## Discussion

The possible pathway proposed for mycotoxicity is the induction of oxidative stress^11^. In vitro and in vivo studies are limited on TeA. Cin was chosen as a prophylactic agent because it is a natural compound and its anti-oxidant and anti-fungal activities have already been reported. In the present study, a murine model has also been developed to test the toxicity caused by TeA.

Oral LD_50_ of tenuazonic sodium salt ranges between 81 and 186 mg/kg bw in mice and rats. In a chicken embryo assay, the LD_50_ for TeA was 548 µg/egg, but TeA did not cause teratogenic effects at doses ranging between 150 and 1500 µg/egg^8,17,18^. Nonetheless, there is a dearth of scientific literature on intraperitoneal LD_50_ of TeA. In the present study, 475 µg/kg BW/day of TeA (PO) and 238 µg/kg BW/day TeA (IP) were used to induce sub-chronic mycotoxicity in the mice model.

The oral route is the most common route of mycotoxin exposure^19^. Since in the present study, TeA producing *Paradendryphiella arenariae* (MW504999) was isolated from tomato rot; hence oral route has been used as one of the routes of TeA administration. IP being the second route of administration was chosen because compounds absorbed intra-peritoneally passes through hepatic circulation before its distribution to other organs^20^. Our study is also in agreement with reports of Al Shoyaib et al. (2019) that IP administration saves the parent compound from its initial break-down phase in the GI tract thus proved to be more toxic than oral administration^21^.

Increasing the intake of vitamins, antioxidants and anti-carcinogenic substances in diet and reduction of mycotoxin levels in food can prevent mycotoxin induced toxicity^22^. Some commonly used treatments for mycotoxicity are glutathione, antioxidants, antifungals, and sequestering agents like cholestyramine, charcoal, clay, antioxidants and probiotics^23^. In the present study, Cin was used as a prophylactic agent for TeA-induced mycotoxicosis in mice. Cin has been reported to have LD_50_ value of 460 mg/kg in mice when given intra-peritoneally^24^. In white mongrel mice, LD_50_ value of Cin was observed to be 2.318 g/kg when administered through the IP route^25^. But in this study, 210 mg/kg body weight of Cin *per os* was found effective against mycotoxicosis induced by TeA in mice. In another study, carried out by Neelabh and Singh (2020), 312 mg/kg Cin on oral administration showed anti-cryptococcal activity in mice^15^.

The acute T-2 toxicity and chronic AFB1 intoxication cause neutrophilia^26^ whilst progressive leukopenia, granulocytopenia and lymphocytosis are the signs of trichothecene mycotoxicoses^19^. The results of this study also documented lymphocytosis, monocytosis, basophilia and eosinophilia concomitant with neutropenia in the PO group while neutropenia, lymphocytosis and monocytosis were observed in the IP group.

Suppression of weight gain in chickens has been reported by Griffin and Chu (1983) when TeA was administered at an increasing dose from sub-lethal to lethal concentrations^18^. Chronic exposure of deoxynivalenol (DON) in the diet has also resulted in the repression of feed consumption and weight gain in experimental animals^27–29^. The low relative weight gain and low average daily gain (kg/day) have also been reported by Alizadeh et al. (2015) when feed contaminated with DON was fed to piglets^30^. But in this study, an inverse relationship was observed between feed intake and weight gain in the MI groups while in prophylaxis groups (POT and IPT), an increase in feed consumption along with suppression in weight gain was recorded.

Organ weight analysis is an important endpoint in toxicological studies to check the harmful effects of xenobiotics and toxic compounds^31,32^. Therefore, in this study weight of organs relative to the body weight of mice was studied. No significant changes in the relative weight of liver and lungs were observed but TeA toxicity caused a significant lowering in renal mass of the MI groups. Changes in the weight of kidney can be a reflection of renal toxicity, chronic progressive nephropathy and renal atrophy^33^. The antioxidant effect of Cin on the kidneys in a time- and dose-dependent manner has been reported^34^. Likewise in our study, anti-oxidant activity of Cin lowered the OS in kidneys of treatment groups. DON and zearalenone (ZEN) are associated with necrosis of the lining of stomach and upper small intestine^35^. The weight of stomach decreased significantly in the MI groups also in this study. Cin has been reported to inhibit gastric inflammatory cytokines, oxidative stress markers and regulate GSH/GSSG ratio^36^. Spleen is an immune organ. During inflammation, huge recruitment of blood cells in inflamed organs leaves spleen with lesser number of blood cells leading to a decrease in splenic weight^37,38^. In the current study also the weight of spleen reduced remarkably in the IP groups. Toxicological studies on T-2 mycotoxin showed increased relative weights of liver and mesenteric lymph node whilst increased relative weight of liver and spleen was recorded when OTA was fed to poultry^39–41^. On the other hand, T-2 toxin in broilers caused decreased relative weight of spleen^42^. Cin has been shown to significantly decrease the relative weights of liver and kidney^43^. In this study, an increase in the spleen weight was observed in treatment groups when compared to their respective MI groups. Cardiac toxicity might be a reason behind the decreased relative weight of heart in the MI groups. The toxic condition can cause arrhythmias which may lead to heart failure^44^. When fuminosin B1 with and without aflatoxin B1 (AFB_1_) was fed to piglets, the heart/body weight ratio increased significantly^45^ The protective role of cinnamaldehyde against cardiovascular diseases has already been reported^46^. Cin treatment in this study also maintained the heart/body weight index but only in the POT group. The relative weight of brain dropped significantly in the MI groups in this study. Mycotoxins such as enniatin B, beauvericin, and aflatoxin are lipophilic molecules and are known to get deposited in the brain by crossing the blood–brain barrier^47^. TeA induced neurotoxicity has not been previously reported. However, intra-nasal exposure of satratoxin G in mouse model was observed to cause destruction of the olfactory sensory nerves^48^. The neuroprotective effect of natural Cin and its derivatives was reported by Yang and Zhou in 2019 and Fu et al. in 2017^49,50^. The relative weight of brain declined sharply in both the mycotoxicosis induced groups. Cin treatment again showed a protective role against the TeA toxin as relative brain weight increased in both the treatment groups.

Interaction of mycotoxins with gut microbiota plays an important role in the progression of mycotoxicosis, particularly hepatocellular carcinoma (HCC)^11^. Likewise, the signs of HCC have been shown in the liver of the MI group as well. No significant change in the weights of the liver of the mycotoxicosis induced groups was noted. Similar were the results of Sun et al. (2014) who fed mice orally with AFB_1_ or ZEA alone or a combination of AFB_1_, ZEA and DON and Bergsjø et al. (1993), who fed mice with DON contaminated oats^51,52^. However, Jiang et al. (2011) and Yarru et al. (2009) reported that exposure to AFB1 and ZEA increase liver weight^53,54^.

The lungs showed oedema, inflammation and hyperplasia. Inflammation might be a result of TeA induced tissue damage^24^. Oedema is often accompanied by increased weight of the lungs. A slight change in the relative weight of lungs was noted in the MI groups which was insignificant.

Kidneys, liver and other affected organs showed gross lesions, blood vessel congestion and haemorrhage when broilers were fed with TeA^8^. Similarly, a gross lesion on the kidney surface and haemorrhage in the renal tissues were observed in MI groups. Besides, cortical vacuolization, haemorrhage and inflammation were also observed. As cellular infiltration was accompanied with haemorrhage and tissue damage, the pathology was indicative of inflammation^24^.

The spleen evidenced haemorrhage, red pulp hyperplasia and apoptotic cells consistent with the results of Giambrone et al.^8^. MI groups exhibited decrease in splenic weight which was further supported by OS and histological studies. The oral administration of Cin either restored the altered enzymes such as ALT, AST, SOD, CAT and MDA levels to near normal or protected the cells from oxidative stress. Thus, the results indicate the antioxidant property of Cin.

The decreased weight and presence of tumours on the surface of the stomach of the MI mice suggested TeA induced gastric toxicity. Histological studies of the stomach revealed cytoplasmic vacuolation and ulceration accompanied by atypical hyperplasia characterized by cellular atypia, abnormal differentiation, and disorganized mucosal architecture^24^. TeA induced pre-cancerous changes have earlier been reported in the oesophageal mucosa of mice^55^. But, this study documented pre-cancerous changes in the stomach of MI groups. The biochemical tests of the MI group also showed generation of ROS leading to cytotoxicity. The prophylaxis groups, however, did not exhibit any sign of oxidative stress.

Giambrone et al. (1978) performed experiments to study the effect of TeA on young chickens. They reported that TeA administration by daily oesophageal intubation resulted in haemorrhage on the surface of the heart^8^. Cyst and haemorrhage along with inflammation in the ventricular region of the heart were noted in the present study also. Myocardial haemorrhage along with macrophage infiltration may be signs of vessel damage or constriction of vessels of small arteries or myocytic toxicity^24^.

AST and ALT levels are elevated in viral and liver disease associated with hepatic necrosis and other disorders^56^. Conversely in this study, a drop in the level of AST in brain and heart (IP, PO) was noticed. The results corroborated the studies of Sun et al. (2014) stating that the administration of AFB1 and ZEA decreased AST (40%) in the serum of mice^51,57^.

Liver is the first organ to interact with toxin when administered intra-peritoneally. In 2010, Theumer et al. observed an increase in the MDA levels due to fumonisins and aflatoxin B_1_ toxicity in the spleen mononuclear cells of male Wistar rats^58^. The impairment of the hepatic antioxidant defense system leads to abnormal functionality of MDA^59^. Due to TeA toxicity, MDA could not regulate the generation of ROS. Thus instead of elevation, its level dropped in IP group. Increased MDA results in an increase in lipid peroxidation in the cell membrane and accelerate cell death^60^. The enhanced activity of MDA led to irreparable damage in the spleen (MI, POT and IPT), heart (PO) and liver (IPT) and can be authenticated by the concurrent studies on Caspases-3. Similar to the studies carried out by Rukmini et al. (2004), decreased SOD, CAT, AST and increased MDA in the brain tissue showed the presence of OS^61^. Because oxidative stress is known to play a significant role in inducing apoptosis directly and indirectly, the cell apoptotic enzyme Caspase-3 was also assessed^62,63^.

Apoptosis is generally associated with the mycotoxicity^64^. Caspases (cysteine-dependent aspartate protease) has a key role in the process of apoptosis^65^. It also takes part in the regulation of the host response during infection with bacteria, viruses and parasites^66^. The activity of caspase-3 was chosen as cell apoptotic marker because its elevated level masks the activity of other caspases in the cell lysates^67^. Caspase-3 increase apoptosis by inhibiting uncontrolled cell division and accumulation of mutations^68^. Cin treatment induced apoptosis by increasing Caspase-3 in the prophylactic groups. Cin induced apoptosis by ROS production in human promyelocytic leukemia cells^69^. Cin has also been reported to induce apoptosis in human hepatoma PLC/PRD/5^70^. In Cin treated groups, liver (POT, IPT), lungs (POT), kidney (POT), stomach (POT, IPT), brain (POT, IPT) showed high activity of caspase 3 activity.

The evidences from animal models and human epidemiological data prove that mycotoxins pose a prominent threat to humans and animals. This study is the first of its kind where a murine model has been developed for sub-chronic TeA induced mycotoxicity via two different routes, per os and IP and also the first report demonstrating the prophylactic activity of Cin in TeA induced mycotoxicosis in vivo. Such knowledge potentially drives the development of novel and innovative strategies for the prevention of mycotoxin contamination and therapy of mycotoxicoses.

## Methods

### Experimental animal

**Figure 12:**
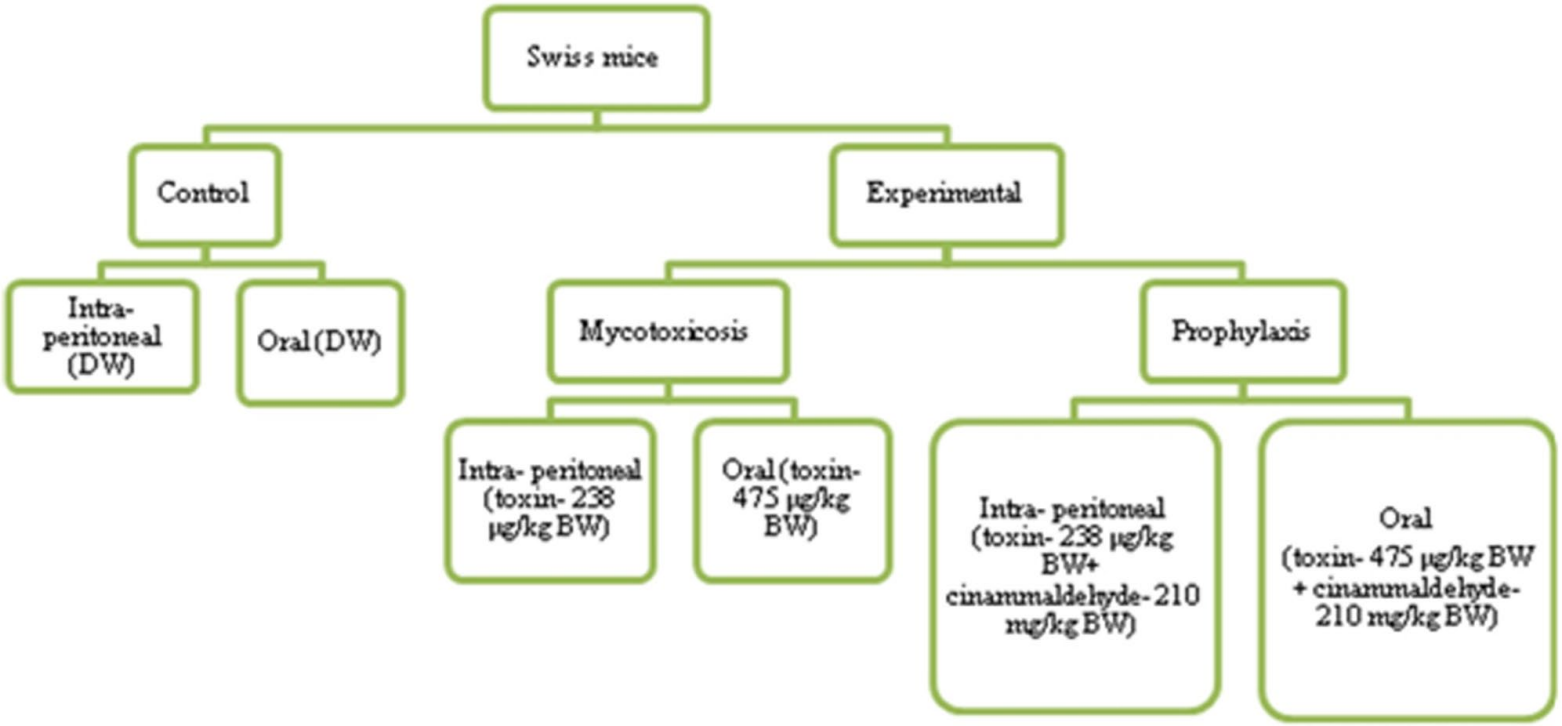
Flowchart of experimental design.

Swiss albino male mice (C3HHC strain) were chosen as experimental model. Two routes of administration-oral (PO) and intra-peritoneally (IP) were used. Mice were divided into 6 groups. The control groups received distilled water while mycotoxicosis-induced groups (PO, IP) were administered TeA orally and intra-peritoneally. Two treatment groups were also set which received TeA as well as the treatment of Cin. These groups were named as POT-the one which received TeA through oral administration and Cin *per os* while the other IPT-which received TeA through intra-peritoneal route and Cin *per os* (Fig. 12). Each group contained 5 animals (n = 5) housed in different cages and fed with commercial rodent diets for 8 weeks. The food ration was replaced daily, and the weights of food portions given and uneaten after 24 h were determined. Weights of the mice were also recorded every 24 h. All experimental protocols were approved by Institutional Animal Ethical Committee, Banaras Hindu University, India (BHU/DoZ/IAEC/2018-19/048). All experiments were performed in accordance with the guidelines and regulations of Institutional Animal Ethical Committee, Banaras Hindu University, India. Euthanasia was performed by sedating the mice with 0.1 ml/20gm IP injection containing a mixture of Ketamine/Xylazine (17.5 mg/ml Ketamine/2.5 mg/ml Xylazine). Thereafter, cervical dislocation was performed within 15 min of sedation^71^. The study was carried out in compliance with the ARRIVE guidelines.

### TeA induced mycotoxicity

TeA isolated from *Paradendryphiella arenariae* (MW504999) cultures (source-tomato) was used for the induction of mycotoxicosis^72^. TeA was characterized using TLC, HPLC and ESI–MS (supplementary data for HPLC provided in Table S1). Distilled water was used as a vehicle in both the routes. Mice were administered with 475 µg/kg/day (PO) and 238 µg/kg/day (IP) of TeA daily for 56 days following Yekeler et al.^55^. Since little is known about the toxicity of TeA, two routes of mycotoxin administration have been used (oral and intra-peritoneal) in order to analyse and compare the damage caused^73^.

### Determination of Cinnamaldehyde dose

Three doses of Cinnamaldehyde (Himedia, GRM3277), 105 mg/kg/day bw (low), 210 mg/kg bw (intermediate), and 420 mg/kg bw (high) were taken into consideration for prophylaxis^74^. Distilled water was used as a vehicle for Cin administration.

### Cinnamaldehyde prophylaxis

Cin administration (orally) was started in the third week of experimentation. After that, TeA and Cin was administered simultaneously throughout the experiment. At the end of the experiment, the mice were euthanized and the organs were aseptically removed. The in vivo toxicity of TeA and the prophylactic effect of Cin were evaluated. The results of these experiments were correlated with biomarkers of cellular oxidative stress.

### Relative weight of organs^75^

The liver, lungs, kidney, spleen, stomach, heart and brain were removed and relative weight of organs was calculated as a percentage of the body weight.

## Enzymatic assays

### Tissue homogenate (TH) preparation and estimation of different parameters

The organs were immediately perfused with ice-cold saline (0.85% sodium chloride) after removal. The tissues (10% w/v) were homogenized in chilled phosphate buffer saline (PBS-0.1 M, pH 7.4). The lysates were centrifuged at 12,000 rpm for 30 min at 4 °C. The supernatant was collected after discarding the pellet (containing cell debris) to carry out various enzymatic assays.

### Estimation of superoxide dismutase (SOD)^76^

Ten µl TH was added to 140 µl cocktail containing 111 µl phosphate buffer, 7.5 µl α-methionine, 4 µl triton X, 7.5 µl hydroxyamine hydrochloride and 10 µl EDTA. The reaction mixture was incubated in a lightbox for 5 min in a 96-well plate. Riboflavin (8 µl) was then added to the mixture and further incubated for 10 min. Following incubation, 100 µl Greiss reagent (50 µl of 0.1% naphthyethylene diamine in DW + 50 µl of 1% sulphanilamide in 5% orthophosphoric acid) was added. TH was replaced by DW in the blank and control; riboflavin was replaced with DW in the blank. Blank was set at 543 nm and absorbance was recorded spectrophotometrically for the control and experimental samples.

### Estimation of malondialdehyde (MDA)^77^

Twenty µl TH was mixed with 20 µl butylated hydroxyl toluene (0.06%). Twenty µl sodium lauryl sulfate (8%) was then added followed by 150 µl thiobarbituricacid (0.8%) and 40 µl DW. The reaction mixture was mixed properly and incubated at 98 °C for an hour in a water bath. On the appearance of pink colour, the mixture was cooled; 100 µl DW and 500 µl butanol:pyridine (15:1) was added. The supernatant was transferred to a 96-well plate and OD was measured against blank at 532 nm.

### Estimation of catalase (CAT)^78^

Fifty µl of the TH was mixed with 600 µl of 15 mM hydrogen peroxide (H_2_O_2_) in a cuvette. The kinetics of the decrease in light absorbance at 240 nm (H_2_O_2_ decomposition) was determined for 3 min. A cuvette containing only PBS served as a blank.

### Estimation of alanine transaminase (ALT) and aspartate aminotransferase (AST)^79^

ALT and AST were analyzed spectrophotometrically using commercially available enzymatic kits (SPAN Auto-diagnostics).

### Caspase 3 activation^80^

The investigation of the Caspase-3 enzyme was carried out using colorimetric caspase-3 (Sigma) assay. The reaction mixture (100 µl) comprised of cell lysate (30 µl), caspase-3 substrate acetyl-Asp-Glu-Val-Asp-p-nitroanilide (final concentration 200 µM), and sodium phosphate buffer (50 mM, pH 7) in a 96-well plate. The reaction mixture was incubated for 90 min at 37 °C in a water bath. Absorbance was measured at 405 nm.

Each assay was carried out in triplicate. The average of both the vehicle (DW) control groups (PO and IP) has been used in the control group value.

### Histological investigation

Tissue samples were removed, rinsed in physiological saline and then fixed in 10% neutral buffered formalin. The fixed tissues were dehydrated in graded ethyl alcohol, rinsed in xylene and then embedded in paraffin wax. Six μm thick sections were serially cut using a Leica Rotary Microtome (Model RM 2125RT; Leica, Microsystems) and mounted on cleaned glass slides. Tissue sections were de-paraffinized in xylene and then hydrated in graded ethyl alcohol in descending concentrations. The sections were stained with Ehrlich's haematoxylin and eosin (HE), dehydrated in graded ethyl alcohol in ascending concentrations, cleared in xylene and mounted with DPX (Distrene dibutyl phthalate xylene). Photomicrogaphy of the sections was performed using Leica DM 2000 digital camera.

### Differential leukocyte count

Blood was collected from euthanized mice of the experimental and control groups by cardiac puncture and thin smears were prepared. The smears were stained with Leishman’s stain for differential leukocyte count.

### Statistical analysis

For each group in each experiment, estimations were based on the data obtained from five mice. Data have been expressed throughout as mean ± SE. Statistical differences between control and experimental groups at each interval were analysed using analysis of variance (ANOVA) followed by Dunnett's and LSD post hoc test. Statistical analyses were conducted using Statistical Package for the Social Sciences (SPSS) for windows (standard version 25) software. Differences were considered statistically significant when p < 0.05.

## Supplementary Information

Supplementary Information.

## References

[1] Ostry V (2008). *Alternaria* mycotoxins: An overview of chemical characterization, producers, toxicity, analysis and occurrence in foodstuffs. World Mycotoxin J..

[2] Gil-Serna J, Vázquez C, González-Jaén MT, Patiño B (2014). Mycotoxins. Toxicology.

[3] Schobert R, Schlenk A (2008). Tetramic and tetronic acids: An update on new derivatives and biological aspects. Bioorg. Med. Chem..

[4] Shigeura HT, Gordon CN (1963). The biological activity of tenuazonic acid. Biochemistry.

[5] Patriarca A, Vaamonde G, Pinto VF, Robinson RK (2014). Alternaria in Encyclopedia of Food Microbiology.

[6] Lurie A, Katz J, Ludwin SK, Seftel HC, Metz J (1969). Platelet life-span and sites of platelet sequestration in onyalai. Br. Med. J..

[7] Fraeyman S, Croubels S, Devreese M, Antonissen G (2017). Emerging *Fusarium* and *Alternaria* mycotoxins: Occurrence, toxicity and toxicokinetics. Toxins.

[8] Giambrone JJ, Davis ND, Diener UL (1978). Effect of tenuazonic acid on young chickens. Poult. Sci. J..

[9] Shokrzadeh M, Ahmadi A, Naghshvar F, Chabra A, Jafarinejhad M (2014). Prophylactic efficacy of melatonin on cyclophosphamide-induced liver toxicity in mice. Biomed. Res. Int..

[10] Omar HEM, Makun HA (2013). Mycotoxins-induced oxidative stress and disease. Mycotoxin and Food Safety in Developing Countries.

[11] Liew WPP, Mohd-Redzwan S (2018). Mycotoxin: Its impact on gut health and microbiota. Front. Cell. Infect. Microbiol..

[12] Bennett JW, Klich M (2003). Mycotoxins. Clin. Microbiol. Res..

[13] Whitlow, L. W. & Hagler, W. M. Mycotoxins in dairy cattle: Occurrence, toxicity, prevention and treatment. *In: Proceedings of Southwest Nutrition Conference*. 124–138 (2005).

[14] Mishra AK, Mishra A, Kehri HK, Sharma B, Pandey AK (2009). Inhibitory activity of Indian spice plant *Cinnamomum zeylanicum* extracts against *Alternaria solani* and *Curvularia lunata*, the pathogenic dematiaceous moulds. Ann. Clin. Microbiol. Antimicrob..

[15] Neelabh SK (2020). Evaluation of antifungal activity of cinnamaldehyde against *Cryptococcus neoformans* var. grubii. Folia Microbiol..

[16] OuYang Q, Duan X, Li L, Tao N (2019). Cinnamaldehyde exerts its antifungal activity by disrupting the cell wall integrity of *Geotrichum citri-aurantii*. Front. Microbiol..

[17] Smith ER, Fredrickson TN, Hadidian Z (1968). Toxic effects of the sodium and the N, N'-dibenzylethylenediamine salts of tenuazonic acid (NSC-525816 and NSC-82260). Cancer Chemother. Rep..

[18] Griffin GF, Chu FS (1983). Toxicity of the *Alternaria* metabolites alternariol, alternariol methyl ether, altenuene, and tenuazonic acid in the chicken embryo assay. Appl. Environ. Microbiol..

[19] Peraica M, Domijan AM (2001). Contamination of food with mycotoxins and human health. Arhiv Higijenu Rada I Toksikologiju.

[20] Koch MA (2006). Experimental modeling and research methodology. The Laboratory ***Rati.

[21] Al Shoyaib A, Archie SR, Karamyan VT (2019). Intraperitoneal route of drug administration: Should it be used in experimental animal studies?. Pharm. Res..

[22] Creppy EE (2002). Update of survey, regulation and toxic effects of mycotoxins in Europe. Toxicol. Lett..

[23] Hope J (2013). A review of the mechanism of injury and treatment approaches for illness resulting from exposure to water-damaged buildings, mold, and mycotoxins. Sci. World J..

[24] National Toxicology Program (2004) Toxicology and carcinogenesis studies of trans-cinnamaldehyde (microencapsulated) in F344/N Rats and B6C3F1 mice, in: NTP TR 514, NIH Publication No. 04-4448.

[25] Shreaz S (2016). Cinnamaldehyde and its derivatives, a novel class of antifungal agents. Fitoterapia.

[26] Weekley LB, Llewellyn GC, O’Rear CE, Llewellyn GC (1989). Altered Differential Leukocyte Counts Induced by Acute and Chronic Administration of Trichothecene T-2 ***Toxin or Aflatoxin B1 in *Biodeterioration Research* 2.

[27] Pestka JJ (2007). Deoxynivalenol: Toxicity, mechanisms and animal health risks. Anim. Feed Sci. Technol..

[28] Dänicke S, Goyarts T, Valenta H (2007). On the specific and unspecific effects of a polymeric glucomannan mycotoxin adsorbent on piglets when fed with uncontaminated or with *Fusarium* toxins contaminated diets. Arch. Anim. Nutr..

[29] Dänicke S, Valenta H, Kersten S (2012). Humic substances failed to prevent the systemic absorption of deoxynivalenol (DON) and its adverse effects on piglets. Mycotoxin Res..

[30] Alizadeh A, Braber S, Akbari P, Garssen J, Fink-Gremmels J (2015). Deoxynivalenol impairs weight gain and affects markers of gut health after low-dose, short-term exposure of growing pigs. Toxins.

[31] Sellers RS, Mortan D, Michael B, Roome N, Johnson JK, Yano BL, Perry R, Schafer K (2007). Society of Toxicologic Pathology position paper: Organ weight recommendations for toxicology studies. Toxicol. Pathol..

[32] Bailey SA, Zidell RH, Perry RW (2004). Relationships between organ weight and body/brain weight in the rat: What is the best analytical endpoint?. Toxicol. Pathol..

[33] Greaves P (2011). Histopathology of Preclinical Toxicity Studies: interpretation and Relevance in Drug Safety Evaluation.

[34] Gowder SJT, Devaraj H (2006). Effect of the food flavour cinnamaldehyde on the antioxidant status of rat kidney. Basic Clin. Pharmacol. Toxicol..

[35] Zain ME (2011). Impact of mycotoxins on humans and animals. J. Saudi Chem. Soc..

[36] Sampath C, Wilus D, Tabatabai M, Freeman ML, Gangula PR (2021). Mechanistic role of antioxidants in rescuing delayed gastric emptying in high fat diet induced diabetic female mice. Biomed. Pharmacother..

[37] Zhou X, Lu Q, Kang X, Tian G, Ing D, Yang J (2021). Protective role of a new polysaccharide extracted from *Lonicera japonica* thunb in mice with ulcerative colitis induced by dextran sulphate sodium. BioMed. Res. Int..

[38] Guo TL, White KL, Charlene M (2010). Methods to assess immunotoxicity. Comprehensive Toxicology (Second edition)< vol*** 5.

[39] Nayakwadi S (2020). Toxicopathological studies on the effects of T-2 mycotoxin and their interaction in juvenile goats. PLoS One.

[40] Gibson RM, Bailey CA, Kubena LF, Huff WE, Harvey RB (1989). Ochratoxin A and dietary protein: 1 Effects on body weight, feed conversion, relative organ weight, and mortality in three-week-old broilers. Poult. Sci. J..

[41] Ayhan F, Yurdakok-Dikmen B, Kuzukiran O, Sireli UT, Manaf M (2017). Mycotoxins in poultry. Poultry Science.

[42] Manafi M, Pirany N, Ali MN, Hedayati M, Khalaji S, Yari M (2015). Experimental pathology of T-2 toxicosis and mycoplasma infection on performance and hepatic functions of broiler chickens. Poult. Sci..

[43] Ahmad RA, Serati-Nouri H, Majid FAA, Sarmidi MR, Aziz RA (2015). Assessment of potential toxicological effects of cinnamon bark aqueous extract in rats. Int. J. Biosci. Biochem. Bioinform..

[44] Iarussi D, Indolfi P, Casale F, Coppolino P, Tedesco MA, Tullio MDT (2001). Recent advances in the prevention of anthracycline cardiotoxicity in childhood. Curr. Med. Chem..

[45] Dilkin P, Zorzete P, Mallmann CA, Gomes JDF, Utiyama CE, Oetting LL, Corrêa B (2003). Toxicological effects of chronic low doses of aflatoxin B1 and fumonisin B1 containing *Fusarium moniliforme* culture material in weaned piglets. Food Chem. Toxicol..

[46] Rao PV, Gan SH (2014). Cinnamon: A multifaceted medicinal plant. Evid. Based Complement Alternat. Med..

[47] Rodríguez-Carrasco Y, Heilos D, Richter L, Süssmuth RD, Heffeter P, Sulyok M, Kenner L, Berger W, Dornetshuber-Fleiss R (2016). Mouse tissue distribution and persistence of the food-born fusariotoxins Enniatin B and Beauvericin. Toxicol let..

[48] Islam Z, Amuzie CJ, Harkema JR, Pestka JJ (2007). Neurotoxicity and inflammation in the nasal airways of mice exposed to the macrocyclic trichothecene mycotoxin roridin a: Kinetics and potentiation by bacterial lipopolysaccharide coexposure. Toxicol Sci..

[49] Yan F, Yang P, Zhao Y, Zhang L, Zhang Z, Dong X, Wu Z, Xu Y, Chen Y (2017). trans-Cinnamaldehyde inhibits microglial activation and improves neuronal survival against neuroinflammation in BV2 microglial cells with lipopolysaccharide stimulation. Evid. Based Complement Alternat. Med..

[50] Qiao-Qiao Y, Jia-Wei Z (2019). Neuroinflammation in the central nervous system: Symphony of glial cells. Glia.

[51] Sun LH (2014). Hepatotoxic effects of mycotoxin combinations in mice. Food Chem. Toxicol..

[52] Bergsjø B, Langseth W, Nafstad I, Jansen JH, Larsen HJS (1993). The effects of naturally deoxynivalenol-contaminated oats on the clinical condition, blood parameters, performance and carcass composition of growing pigs. Vet. Res. Commun..

[53] Jiang SZ (2011). Effects of purified zearalenone on growth performance, organ size, serum metabolites, and oxidative stress in postweaning gilts. J. Anim. Sci..

[54] Yarru LP, Settivari RS, Antoniou E, Ledoux DR, Rottinghaus GE (2009). Toxicological and gene expression analysis of the impact of aflatoxin B1 on hepatic function of male broiler chicks. Poul. Sci..

[55] Yekeler H, Bitmiş K, Ozçelik N, Doymaz MZ, Çalta M (2001). Analysis of toxic effects of *Alternaria* toxins on esophagus of mice by light and electron microscopy. Toxicol. Pathol..

[56] Hussein HS, Brasel JM (2001). Toxicity, metabolism, and impact of mycotoxins on humans and animals. Toxicol..

[57] Burtis CA, Ashwood ER, Bruns DE (2012). Tietz textbook of clinical chemistry and molecular diagnostics- ebook. Elsevier Health Sci..

[58] Theumer MG (2010). Subchronic mycotoxicoses in Wistar rats: Assessment of the *in vivo* and *in vitro* genotoxicity induced by fumonisins and aflatoxin B1, and oxidative stress biomarkers status. Toxicology.

[59] Zhan CD, Ram K, Sindhu RK, Pang J, Ehdaie A, Vaziri ND (2004). Superoxide dismutase, catalase and glutathione peroxidase in the spontaneously hypertensive rat kidney: Effect of antioxidant-rich diet. J. Hypertens..

[60] Ayala A, Muñoz MF, Argüelles S (2014). Lipid peroxidation: Production, metabolism, and signaling mechanisms of malondialdehyde and 4-hydroxy-2-nonenal. Oxid. Med. Cell Longev..

[61] Rukmini MS, D’souza B, D’souza V (2004). Superoxide dismutase and catalase activities and their correlation with malondialdehyde in schizophrenic patients. Indian J. Clin. Biochem..

[62] Brahmi F (2017). Evidence of biological activity of Mentha species extracts on apoptotic and autophagic targets on murine RAW264.7 and human U937 monocytic cells. Pharm. Biol..

[63] Chandra D, Choy G, Tang DG (2007). Cytosolic accumulation of Hsp60 during apoptosis with or without apparent mitochondrial release evidence that its pro-apoptotic or pro-survival functions involve differential interactions with caspase-3. J. Biol. Chem..

[64] Doi K, Uetsuka K (2011). Mechanisms of mycotoxin-induced neurotoxicity through oxidative stress-associated pathways. Int. J. Mol. Sci..

[65] Srivastava A, Mistri A, Mittal S, Mittal AK (2020). Alterations in the epidermis of the carp, *Labeo rohita* (Cyprinidae: Cypriniformes), infected by the bacteria, *Aeromonas hydrophila*: A scanning electron microscopic, histopathological and immunohistochemical investigation. J. Fish. Dis..

[66] Nagata S (1997). Apoptosis by death factor. Cell.

[67] Poręba M, Stróżyk A, Salvesen GS, Drąg M (2013). Caspase substrates and inhibitors. Cold Spring Harbor Perspect. Biol..

[68] Cooper GM, Cooper GM (2000). The development and causes of cancer. The Cell: A Molecular Approach.

[69] Ka H (2003). Cinnamaldehyde induces apoptosis by ROS-mediated mitochondrial permeability transition in human promyelocytic leukemia HL-60 cells. Cancer Lett..

[70] Lin LT (2013). Cinnamaldehyde-induced apoptosis in human hepatoma PLC/PRF/5 cells involves the mitochondrial death pathway and is sensitive to inhibition by cyclosporin A and z-VAD-fmk. Anticancer Agent Med. Chem..

[71] University of IOWA. Vertebrate Animal Research. https://animal.research.uiowa.edu/iacuc-guidelines-anesthesia (2020).

[72] Woudenberg JHC, Groenewald JZ, Binder M, Crous PW (2013). *Alternaria* redefined. Stud. Mycol..

[73] Davis JN, Courtney CL, Superak H, Taylor DK (2014). Behavioral, clinical and pathological effects of multiple daily intraperitoneal injections on female mice. Lab. Anim. (NY).

[74] OECD (2009). Organisation for Economic Co-operation and Development. Test no. 453: Combined Chronic Toxicity/Carcinogenicity Studies.

[75] Riaz H, Mahmood F, Khan MZ, Khan A, Muhammad F (2011). Pathological and genotoxic effects of atrazine in male Japanese quail (*Coturnix japonica*). Ecotoxicology.

[76] Das K, Samanta L, Chainy GBN (2000). A modified spectrophotometric assay of superoxide dismutase using nitrite formation by superoxide radicals. Indian J. Biochem. Biophys..

[77] Ohkawa H, Ohishi N, Yagi K (1979). Assay for lipid peroxides in animal tissues by thiobarbituric acid reaction. Anal. Biochem..

[78] Aebi, H. Catalase in *Methods of enzymatic analysis* (ed. Bergmeyer, H. V.) 673–684 (Academic press, 1974).

[79] Reitman S, Frankel S (1957). Determination of glutamate-pyruvate transaminase (ALT) and aspartate aminotransfrase (AST). J. Clin. Pathol..

[80] Srivastava P, Sarma A, Chaturvedi CM (2018). Targeting DNA repair with PNKP inhibition sensitizes radioresistant prostate cancer cells to high LET radiation. PLoS One.

