# Supplementary material for: Evaluation of prophylactic efficacy of cinnamaldehyde in murine model against Paradendryphiella arenariae mycotoxin tenuazonic acid-induced oxidative stress and organ toxicity
Source: Sci Rep. 2021 Sep 30;11:19420. doi: 10.1038/s41598-021-98319-8 (PMC8484465; doi:10.1038/s41598-021-98319-8)
Supplement: Supplementary file 1 — Supplementary Information. [file 41598_2021_98319_MOESM1_ESM.pdf]

Evaluation of Prophylactic Efficacy of Cinnamaldehyde in Murine model against *Paradendryphiella arenariae* mycotoxin Tenuazonic acid-induced oxidative stress and organ toxicity

Ankita Kumari, Karuna Singh*

Animal Mycology Laboratory, Department of Zoology, Mahila Mahavidyalaya, Banaras Hindu University, Varanasi-221005

***Corresponding author**
Dr. Karuna Singh
Professor,
Department of Zoology,
Mahila Mahavidyalaya,
Banaras Hindu University
Varanasi-221005
E-mail id- karunasingh5@gmail.com
Contact- +919335416923

Supplementary data

Quantitative detection of TeA

HPLC

In quantitative analyses, standard TeA sample (1000 µg/ml in methanol) was diluted in 3 different concentrations of 200, 100, 10 µg/ml and a volume of 20 µl was injected for HPLC analysis. The peak area from the responding peak was integrated using on-system tools provided by Empower 3. At least 4 injections were made for each concentration, and the peak areas were then plotted against the absolute amounts of TeA used to obtain a standard curve.

Result

Table S1- Quantitative assay of TeA by HPLC (Wavelength- 250 nm)

Concentration (µg/ml)	10	100	200	1000	TeA Extract
Height	3554	33495	60280	510678	8629
Area	382628	3367469	8587942	43116529	483006
RT (min)	7.074	7.048	7.265	7.534	8.16

Based on the above results, the concentration of TeA in *P. arenariae* extract was calculated to be 0.412 mg/ml in 43.33 mg/ml of metabolite.
